# Incidence and Causes of Cellulitis Among Patients at Tupua Tamasese Meaole Hospital in Upolu, Samoa in 2019

**DOI:** 10.7759/cureus.48318

**Published:** 2023-11-05

**Authors:** Addisalem Hailu Wondafrash, Uila Laifa Lima, Degu Abebe, Kidus S Negash

**Affiliations:** 1 Department of Medicine, Oceania University of Medicine, Apia, WSM; 2 Department of Medicine, Hayat Medical College, Addis Ababa, ETH

**Keywords:** incidence of cellulitis, cellulitis in samoa, mean age of cellulitis, sex differences in cellulitis, common site of cellulitis, causes of cellulitis, flucloxacillin, staphylococcus and streptococcus, cellulitis

## Abstract

Objective:To study the incidence and causes of cellulitis in patients who visited the only tertiary hospital in Samoa, i.e., Tupua Tamasese Meaole (TTM) Hospital, in 2019.

Method: Of the total of 14,198 patients who presented to TTM Hospital in 2019, a chart review of all 258 patients who presented with cellulitis was conducted*.* All charts with the final primary admitting diagnosis of cellulitis were extracted. No exclusion criteria were employed, and raw data were analyzed manually.

Results: Of the 14,198 patients who sought care at TTM Hospital in 2019, 258 patients received care for cellulitis. This represents an incidence rate of 1.8%. Most patients were male (62.4%). Those in the age group of 41 to 80 years old accounted for 79.5% of the total. The leg (94.6%) was the major site of infection. Of those who had blood cultures drawn, 76.4% had negative results. Of the 56 patients with positive microbial growth, *Staphylococcus* and *Streptococcus* species accounted for a combined total of 71.4% of the cases. The mainstay of antibiotic treatment was flucloxacillin alone or in conjunction with other antibiotics (92.2%). Of the many comorbidities affecting patients, diabetes (44.2%) was the most prevalent. Hospital admission, ranging from three to 10 days was needed in 63.5% of patients.

Conclusion: The incidence rate of cellulitis at TTM in 2019 was 1.8%, which was marginally higher than noted in other parts of the world. Male patients and people over the age of 40 years are affected the most. The leg is affected the most mainly by *Staphylococcus* and *Streptococcus* species. Flucloxacillin is the main antibiotic used to treat cellulitis at the TTM Hospital. From the data analyses, it is inferred that a large proportion of patients who presented had moderate to severe cellulitis.

## Introduction

Cellulitis is a common medical condition that is caused when the skin’s barrier mechanism is compromised and allows infectious agents to enter deeper tissues. Patients with cellulitis can present with mild, moderate, or severe forms of cellulitis. Mild cellulitis is characterized by signs of erythema and edema, without signs of systemic toxicity like fever, chills, and hypotension [1]. Moderate cellulitis is known to present with fever, increased inflammatory markers, and signs of painful erythema with some skin infections at a deeper level [2]. Patients with severe cellulitis present with fever, tachycardia, hypovolemic shock, and deeper soft tissue, skin, and limb-threatening infections due to vascular compromise. Moderate and severe types of cellulitis were noticed to be common in developing countries and people with poor socioeconomic conditions [3]. If not treated in a timely manner, cellulitis may lead to disabilities and life-threatening medical conditions such as septicemia and osteomyelitis. About 200 cases of cellulitis per 100,000 patients are registered worldwide per year [4]. Although patients with comorbidities, immunocompromise, or lower socio-economic status have a higher risk of cellulitis, it may also occur in healthy individuals with no predisposing conditions.

It is found that cases of cellulitis increase during humid and hot weather (tropical climates), implicating that climate has an impact on the pathogenesis of cellulitis [5]. As average monthly temperatures increase, so does the risk for admissions due to cellulitis, even after controlling for patient age, sex, type of payer, length of stay, comorbidities, geographic region, latitude, and longitude [6]. Hospital admission rates for cellulitis in New Zealand are twice than that of Australia and the United States. Pacific people are at least 1.5 times more likely to have a diagnosis of cellulitis compared to other countries [7].

Predisposing risk factors for cellulitis in the Samoan population are the prevalence of type 2 diabetes, increase in BMI, and inclination to treat skin ailments by using traditional healers. In 2016, the most recent year that is available, the prevalence of type 2 diabetes mellitus within the Samoan population was projected to be 26% in both men and women in 2020 [8]. It was also projected that obesity will be prevalent in 59% of Samoan men and 81% of Samoan women in 2020 [8]. Patients present late in the course of their infectious process due to a strong desire to adhere to traditional practice without medical interference [9]. It is common for patients to seek medical care only after experiencing systemic manifestations of the infection. As a result, Samoa is an ideal country to study moderate to severe cellulitis due to its tropical climate, high prevalence rate of type 2 diabetes mellitus and obesity, and propensity of patients to seek medical attention later in the disease process.

The objective of this research is to investigate the prevalence of cellulitis in patients visiting TTM, determine the etiology and risk factors for the development of cellulitis in these patients, investigate its pathology, analyze the severity of cellulitis cases, examine whether traditional medicine has been used prior to seeking medical care and study its impact on healing, and study types of medications used as treatment.

## Materials and methods

The researchers conducted this study after receiving ethics and human research approval and consent from the Oceania University of Medicine (Approval Number: 20-0821AW) and the Ministry of Health, Health Research Committee of the Samoan Government.

All patients diagnosed with cellulitis who visited Tupua Tamasese Meaole (TTM) Hospital in 2019, aged less than 100 (n = 258), were included in the study, without any exclusion criteria. A retrospective chart review of all 258 patients who presented with cellulitis was conducted. All charts with the final primary admitting diagnosis of cellulitis were extracted. Analyses of the data were done manually and included the proportion of cellulitis based on sex and age groups, the sites (body parts) of cellulitis infection, the type(s) of microbes isolated from blood cultures, the types of antibiotic(s) used for the treatment of cellulitis, proportion of cellulitis cases that required incision and drainages, and the presence or absence of comorbidities.

## Results

Of the 14,198 patients who were seen at TTM Hospital in 2019, charts of 258 patients had an admitting diagnosis of cellulitis, representing 1.8% of total patient visits. Analysis of the 258 patients with cellulitis shows that 161 (62.4%) were male (Table 1).

**Table 1:** Proportion of patients (sex)

Sex	Number of patients	Percentage (%)
Male	161	62.4%
Female	97	37.6%
Total	258	100%

In terms of patients’ age distribution, 102 (39.6%) were between 41 and 60 years old, and 103 (39.9%) were between 61 and 80 years old. In total, those between 41 and 80 years old accounted for most of the cellulitis cases, totaling 79.5% of the total. Nine (3.5%) patients were under 20 years old, and 23 (8.9%) were between 21 and 40 years old. In total, those who were below 40 years of age accounted for 12.4%. Twenty-one (8.1%) patients were between the ages of 81 and 100 years old (Table 2).

**Table 2:** Proportion of patients (age groups)

Age group	Number of patients	Percentage (%)
0-20	9	3.5%
21-40	23	8.9%
41-60	102	39.6%
61-80	103	39.9%
81-100	21	8.1%
Total	258	100%

Of the 258 patients, 244 (94.6%) patients had cellulitis on their legs. The rest 14 (5.4%) patients had cellulitis in other parts of the body (Table 3).

**Table 3:** Body parts affected by cellulitis

Location	Number of patients	Percentage (%)
Leg	244	94.6%
Hand	4	1.6%
Buttock	3	1.1%
Face	3	1.1%
Arm	2	0.8%
Abdomen	1	0.4%
Scalp	1	0.4%
Total	258	100%

Of the 258 patients sampled, 237 (91.9%) patients had blood cultures drawn. Of these, microbes were detected in 56 (23.6%) patients’ blood cultures. *Staphylococcus* species was detected in 25 (44.6%) patients, *Streptococcus* species in 15 (26.7%) patients, mixed organisms in eight (14.3%) patients, *Pseudomonas aeruginosa* and *Acinetobacter *species in three (5.4%) patients each, and *Enterobacter cloacae* in two (3.6%) patients (Table 4).

**Table 4:** Type of microbes isolated ^± ^Out of 56 patients that had positive blood culture results. ^* ^Of which two patients had methicillin-resistant *Staphylococcus aureus*.

Blood culture result	Number of patients	Percentage (%)
No blood culture drawn	21	8.1%
Blood culture drawn	237	91.9%
No growth	181	76.4%
Bacterial growth	56	23.6%
Types of bacteria	Number of patients^±^	Percentage (%)^±^
*Staphylococcus* species^*^	25	44.6%
*Streptococcus* species	15	26.7%
Mixed organisms	8	14.3%
Pseudomonas aeruginosa	3	5.4%
*Acinetobacter* species	3	5.4%
Enterobacter cloacae	2	3.6%
Gross total	258	100%

In terms of antibiotics used to treat patients, flucloxacillin was used in 238 (92.2%) patients. Other antibiotics used included ceftriaxone in 11 (4.3%), amoxicillin-clavulanate in four (1.5%), and other antibiotics such as clindamycin, cephalexin, and penicillin in five (2%) patients (Table 5).

**Table 5:** Antibiotics used to treat patients ^a^ Flucloxacillin alone or in combination with other antibiotics. ^b^ Ceftriaxone alone or in combination with other antibiotics other than flucloxacillin. ^c^ Amoxicillin-clavulanate alone or in combination with other antibiotics other than flucloxacillin and/or ceftriaxone. ^d^ Others include one each for clindamycin, ampicillin, cephalexin, penicillin V, and penicillin G.

Antibiotics	Number of patients	Percentage (%)
Flucloxacillin^a^	238	92.2%
Ceftriaxone^b^	11	4.3%
Amoxicillin-clavulanate^c^	4	1.5%
Others^d^	5	2%
Total	258	100%

Incision and drainage of wounds were done on 59 (22.9%) patients (Table 6).

**Table 6:** Proportion of wound incision and drainage

Incision and drainage	Number of patients	Percentage (%)
No	199	77.1%
Yes	59	22.9%
Total	258	100%

Of the 258 patients, 185 (71.7%) had comorbidities. Diabetes was present in 114 (44.2%), hypertension in 21 (8.1%), cardiac complications in 17 (6.6%), and obesity in eight (3.1%) patients (Table 7).

**Table 7:** Comorbidities of patients ^+ ^Diabetes plus other comorbidities such as hypertension and chronic kidney disease. ^# ^Hypertension plus other comorbidities, excluding diabetes. ​​​​​​​^§ ^Congestive heart failure (seven patients), atrial fibrillation (seven patients), and rheumatic heart disease (three patients). ​​​​​​​^¶ ^Psoriasis, Parkinson’s disease, benign prostatic hyperplasia, cerebral palsy, gallbladder cancer, idiopathic thrombocytopenia, hepatitis C, and colorectal cancer - each with one patient.

Comorbidities	Number of patients	Percentage (%)
None	73	28.3%
Diabetes^+^	114	44.2%
Hypertension^#^	21	8.1%
Cardiac pathologies^§^	17	6.6%
Gout	9	3.5%
Obesity	8	3.1%
Asthma	3	1.2%
Filariasis	3	1.2%
Epilepsy	2	0.7%
Others^¶^	8	3.1%
Total	258	100%

The length of hospital stay ranged from one to 55 days. Fifty-seven (22.1%) patients were admitted for one to two days. The majority (95, 36.8%) were admitted for three to five days, and 69 (26.7%) stayed in hospital for six to 10 days. In total, 164 (63.5%) patients needed admission ranging from three to 10 days. Those who needed admission for more than 10 days but less than 20 days accounted for 28 (10.9%) patients. Four (1.6%) patients needed inpatient treatment lasting 20-30 days, while the remaining five (1.9%) patients were treated as an inpatient for more than 30 days (Table 8).

**Table 8:** Length of hospital admission

Number of days	Number of patients	Percentage (%)
1-2	57	22.1%
3-5	95	36.8%
6-10	69	26.7%
11-20	28	10.9%
20-30	4	1.6%
Greater than 30	5	1.9%
Total	258	100%

## Discussion

This study looked at all cellulitis cases that were diagnosed at TTM in 2019. Of the 14,198 patients who sought care at TTM in 2019, 258 patients were treated for cellulitis. Thus, the incidence rate of cellulitis among patients at TTM in 2019 was 1.8%. This shows a marginally higher rate of cellulitis at TTM, compared, for instance, to 1.4% of cellulitis cases noted in hospital admission in the USA [10]. This difference could be attributed to our cohort including both admitted and non-admitted patients, the social, economic, demographic, climate, and risk factor differences between the two countries, in addition to Samoan patients’ propensity to present late for medical care. The result of this study shows that males were affected with cellulitis more than females. Other researchers have also found that there is a higher incidence of cellulitis in males of all ages compared to females [11]. Of the different age groups of patients that visited TTM Hospital with cellulitis, the majority were in the age group of 41 to 80 years old (79.5%), where the mean age for most cellulitis cases falls at 60.5 years old. Other researchers have also ascertained that cellulitis most often occurs in middle-aged and older adults [12]. The mean age for cellulitis was 63.4 years old [13]. This shows that the mean incidence age of cellulitis in Samoa is comparatively younger.

Our study indicated that the leg was affected the most (94.6%) compared to other parts of the body. Cellulitis usually affects the lower extremities more commonly compared with other parts of the body [14,15]. Of the 258 patients, 237 (91.8%) patients were deemed to have presented with moderate to severe cases of cellulitis to warrant blood cultures drawn. Of these, 56 (23.6%) patients had positive blood culture results. This is in line with the literature that states that the etiologic organisms of cellulitis are detected in only 20% to 30% of blood cultures drawn, signifying a low positivity rate [16]. A combined total of 40 (71.3%) patients had either *Staphylococcus* or *Streptococcus* bacterial species. In general, *Streptococcus pyogenes* and *Staphylococcus aureus* are the two most common causes of cellulitis [17]. The same microbes are also thought to be the “conventional” disease pathogens in lower leg cellulitis [18]. More specifically, most uncomplicated cases of cellulitis are caused by β-hemolytic streptococci and methicillin-sensitive *Staphylococcus aureus *(MSSA) [19]. Despite the climate, high rate of type 2 diabetes, obesity, and late presentation for medical care, the primary etiologic agents of cellulitis in Samoa are the same as in other countries.

In this study, we found that flucloxacillin alone or in combination with other antibiotics was used as a treatment in 92.2% of the cases. Antibiotics with streptococci coverage are the best practice advice for cellulitis [20]. Furthermore, it is mentioned that in areas without methicillin-resistant *Staphylococcus aureus* (MRSA), the antibiotic of choice consists of penicillinase-resistant beta-lactam antibiotics such as flucloxacillin [21]. This is in agreement with our study as most cases in Samoa were MSSA. Fifty-nine (22.9%) patients had incision and drainage of their infection, in addition to antibiotics treatment. Drainage of exudates or abscesses from wounds has been known to help the wound heal and reduce hospital stay. The primary treatment for skin and soft tissue abscesses is incision and drainage, with or without adjunctive antibiotic therapy [22].

Among the many comorbidities present in patients included in our study, three of the most noted comorbidities were diabetes in 114 (44.2%), hypertension in 21 (8.1%), and cardiac complications in 17 (6.6%) patients. Similarly, other researchers have also noted the association of comorbidities, such as hypertension, diabetes, congestive heart failure, and dyslipidemia, with cellulitis [23].

In total, 164 (63.5%) patients were admitted for three to 10 days. Although the reasons that determined longer periods of admission were not apparent on chart review, utilizing deductive reasoning and taking into consideration the proportion of patients that had blood cultures drawn, it is reasonable to assume that most patients must have presented with moderate to severe cases of cellulitis. We postulate that the reason behind the lack of concrete criteria for admission is the universal lack of adequate research that can guide providers’ decisions on when to admit or not [10]. Nonetheless, the Infectious Diseases Society of America states that patients exhibiting systemic signs of infection mostly require inpatient admission [10]. Systemic signs of infection are present in moderate to severe cases of cellulitis.

One of the goals of this research project was to examine whether traditional medication has been used prior to admission and analyze its impact on healing. Unfortunately, due to the lack of such information being documented in patients’ charts, we were unable to examine it. Moreover, we resorted to the application of deductive reasoning to analyze the severity of cellulitis cases that were presented to TTM Hospital in 2019, as such information was not explicitly referred to in patients’ charts.

## Conclusions

The incidence rate of cellulitis in patients who visited TTM Hospital in Samoa in 2019 was found to be marginally higher at 1.8%. Male patients and people over the age of 40 years are affected most frequently. Leg is the most affected mainly by *Staphylococcus *and* Streptococcus* species. Flucloxacillin is the main and first-choice antibiotic used to treat cellulitis at the TTM Hospital. Diabetes is by far the major comorbidity present. Taking into consideration the length of hospital admission and the proportion of patients who had blood cultures drawn, it is reasonable to conclude that a large proportion of patients who presented had moderate to severe cellulitis. As observed by other researchers, the tropical nature of Samoan weather may also be the predisposing factor for the incidence of cellulitis. The impact of weather on the incidence and cause of cellulitis may be of interest for further research.
